# BCAR4 activates GLI2 signaling in prostate cancer to contribute to castration resistance

**DOI:** 10.18632/aging.101664

**Published:** 2018-12-04

**Authors:** Zhiping Cai, Yapei Wu, Yao Li, Jizhong Ren, Linhui Wang

**Affiliations:** 1 Department of Urology, Shanghai Changzheng Hospital, Shanghai 200003, China; 2 Department of Urology, Huashan Hospital, Shanghai 200010, China

**Keywords:** long noncoding RNA, BCAR4, GLI2 pathway, castration-resistant prostate cancer

## Abstract

Long non-coding RNAs (lncRNAs) have been found essential for tumorigenesis of prostate cancer (PC), but its role in the regulation of castration-resistant prostate cancer (CRPC) is poorly identified. Here, we showed that a lncRNA, Breast-Cancer Anti-Estrogen Resistance 4 (BCAR4), which plays a pivotal role in the tamoxifen-resistance of breast cancer, was significantly upregulated in CRPC, but not in castration-sensitive prostate cancer (CSPC), compared to normal prostate tissue. High BCAR4 levels in CRPC were correlated with poor patients’ overall survival. Androgen increased growth and migration of androgen receptor (AR)-positive PC346 cells, which was abolished by the antagonist of androgen. Overexpression of BCAR4 in PC346 cells increased cell growth and migration, but turned the cells insensitive to androgen. On the other hand, growth and migration of AR-negative DU145 cells are insensitive to androgen, while depletion of BCAR4 in DU145 cells not only decreased cell growth, but also turned the cells sensitive again to androgen. Moreover, BCAR4 activated GLI2 downstream genes, and correlated with the levels of these GLI2-target genes in CRPC. Depletion of GLI2 abolished the effects of BCAR4 on cell growth and migration. Together, our data suggest that BCAR4 may activate GLI2 signaling in PC to contribute to castration resistance.

## Introduction

Prostate cancer (PC) is a common malignant cancer that affects aged men [1]. The occurrence and development of PC generally depend on the stimulation of androgen [2]. The local advanced patients, patients with metastatic spread of tumors, and patients who relapse after conventional treatment are currently preferred by clinical endocrine therapy, also known as androgen deprivation therapy (ADT) [2]. ADT Includes castration therapy (surgical castration or drug castration), antiandrogen therapy (bicalutamide or flutamide) or combined castration and antiandrogen therapy [3].

Although the stage that responds to endocrine therapy and the response time may vary from person to person due to the tumor heterogeneity, almost all patients eventually develop hormone-independent PC or castration-resistant prostate cancer (CRPC), when more than 90% of the patients will have bone metastasis of primary tumor with concomitant symptoms like severe pain, pathological fractures, spinal compression, and even intracranial nerve disability and anemia [4]. Patients with castration resistance to PC are usually given chemotherapeutic drugs, but these drugs often cause serious side effects and have a poor improvement of the patients’ survival [5]. Recently, some new drugs have been put into clinical use, such as Cabazitaxel that targets tubulin, Sipuleucel-T that targets immune system, androgen synthesis inhibitor Abiraterone, and the androgen receptor antagonist Enzalutamide, etc [6]. Nevertheless, metastatic CRPC is generally regarded as an incurable disease. Hence, it is important to study the molecular mechanisms underlying the development of CRPC, which remains poorly characterized.

In addition to the well-known protein encoding RNA (mRNA), ribosomal RNA (rRNA) and amino acid transfer RNA (tRNA), there is also a small class of non-coding RNAs (ncRNAs) [7]. According to their size (200 bases as a boundary), ncRNAs are divided into two categories: small non-coding RNA (small molecular RNA (e.g. microRNAs belong to this class) and long non-coding RNA (lncRNA) [7]. Although less than 2% of the sequences in the human genome encode proteins, most other sequences can also be actively transcribed, and have specific functions [7]. These non-coding RNAs usually bind to DNA, RNA, and even proteins to regulate chromatin remodeling, mRNA degradation, RNA splicing and editing, and protein translation [7]. The cancer-associated lncRNA has been shown to be closely related to the process of tumor initiation, proliferation and invasion, but the detailed molecular mechanisms need further study [8]. Specially, some lncRNAs have been shown to control tumorigenesis of PC [9]. With the widespread use of second-generation sequencing technologies in recent years and the increasing annotations of lncRNA gene sequences, researchers found more and more lncRNA genes associated with CRPC [10]. For example, the 8q24 segment on the chromosome is an important PC-related segment, and some genes have SNP mutations that can promote prostate cancer cells from androgen sensitivity to highly malignant castration resistance [10]. There is evidence that several lncRNAs in this segment, such as PCAT1, PRNCR1 and PVT1, are likely to participate into control of this transformation [10].

Breast-Cancer Anti-Estrogen Resistance 4 (BCAR4) is a lncRNA that plays a pivotal role in the tamoxifen-resistance of breast cancer [11]. Previous studies have found that BCAR4 contributes to antiestrogen resistance and promotes breast cancer proliferation and metastasis through regulating noncanonical Hedgehog/GLI2 pathway [12–14]. BCAR4 expression indicates aggressiveness and poor prognosis of human breast cancer [15,16], osteosarcoma [14], non-small cell lung cancer [17], and cervical carcinoma [18]. However, a role of BCAR4 in PC, especially CRPC, has not been reported.

In the present study, we measured the expression of BCAR4, its correlation with clinicopathological characteristics, and its biological roles in castration-sensitive prostate cancer (CSPC) and CRPC. We found that BCAR4 was significantly upregulated in CRPC, but not in CSPC, compared to normal prostate tissue. High BCAR4 levels in CRPC were correlated with poor patients’ overall survival. Among 7 commonly used PC cell lines, androgen receptor (AR)- expressing PC346 expressed the least level of BCAR4, while AR-null DU145 expressed the highest level of BCAR4. Androgen increased PC346 cell growth and migration, which was abolished by the antagonist of androgen. Overexpression of BCAR4 in PC346 cells increased cell growth and migration but turned the cells insensitive to androgen. On the other hand, growth and migration of DU145 cells are insensitive to androgen, while depletion of BCAR4 in DU145 cells not only decreased cell growth, but also turned the cells sensitive again to androgen. Moreover, we found that GLI2 promoter was directly activated by BCAR4 in PC cells. BCAR4 activated GLI2 downstream genes and correlated with the levels of these GLI2-target genes in CRPC. Depletion of GLI2 abolished the effects of overexpression of BCAR4 on cell growth and migration in PC346 cells, while overexpression of GLI2 abolished the effects of depletion of BCAR4 on cell growth and migration in DU145 cells.

## RESULTS

### BCAR4 is upregulated in CRPC and correlated with poor prognosis of patients

**Figure 1:**
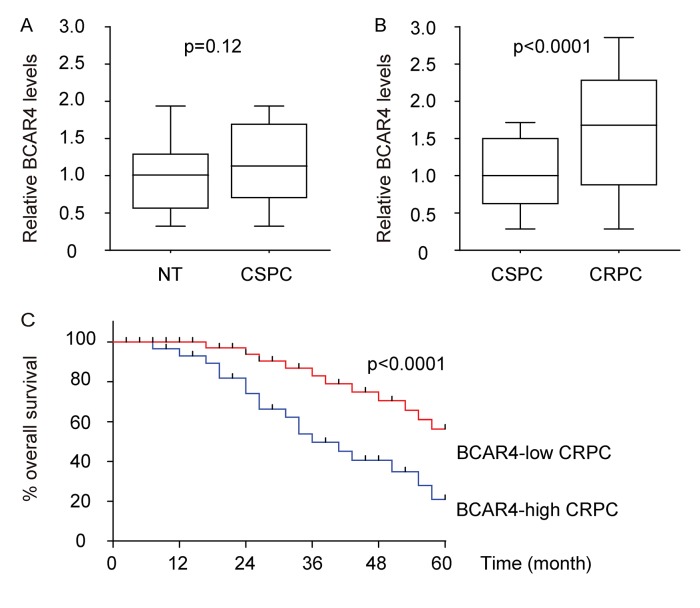
**BCAR4 is upregulated in CRPC and correlated with poor prognosis of patients.** (**A**) BCAR4 levels were examined in 50 pairs of castration-sensitive prostate cancer (CSPC) and paired adjacent normal prostate tissues (NT), by RT-qPCR. (**B**) 50 CSPC and 40 CRPC specimens were assessed by BCAR4 by RT-qPCR. (**C**) The 40 CRPC tissues were classified into two groups using the median value of all cases as the cutoff point. The Kaplan-Meier curves was used to analyze patients’ overall survival.

First, BCAR4 levels were examined in 50 pairs of castration-sensitive prostate cancer (CSPC) and paired adjacent normal prostate tissues (NT), showing no significant difference (Figure 1A). Next, 50 CSPC and 40 CRPC specimens were assessed by BCAR4 (Table 1). We found that BCAR4 was significantly upregulated in CRPC, compared to CSPC and normal prostate tissue (Figure 1B). We analyzed the correlation between BCAR4 levels and clinicopathological characteristics of the 40 CRPC patients. The 40 CRPC tissues were classified into two groups using the median value of all cases as the cutoff point. From the Kaplan-Meier curves, we found that patients’ overall survival was significantly poorer in CRPC with high BCAR4 levels (p<0.0001, Figure 1C). Thus, BCAR4 was significantly upregulated in CRPC, but not in CSPC and high BCAR4 levels in CRPC appeared to be correlated with poor patients’ overall survival.

**Table 1:** Clinicopathological characteristics (total).

	CSPC Patients (n; %)	CRPC patients (n; %)	P value
Age (<65/≥65 years old)	10 (20%) /40 (80%)	5 (12.5%) /35 (87.5%)	0.66
Tumor site (prostate)	50 (100%)	50 (100%)	
Tumor grade (well or moderate/poor)	6 (12%) /18 (36%) /26 (52%)	2 (5%) /8 (20%) /30 (75%)	0.08
Tumor stage (I/II/III/IV)	0 (0%) /5 (10%) /15 (30%) /30 (60%)	0 (0%) /2 (5%) /8 (20%) /30 (75%)	0.14
Lymph node metastasis (yes/no)	42 (84%) /8 (16%)	38 (95%) /2 (5%)	0.09
Distal metastasis at diagnosis (yes/no)	3 (6%) /47 (94%)	15 (37.5%) /25 (62.5%)	0.008

### BCAR4 increases androgen-independent PC cell growth and migration in vitro

**Figure 2:**
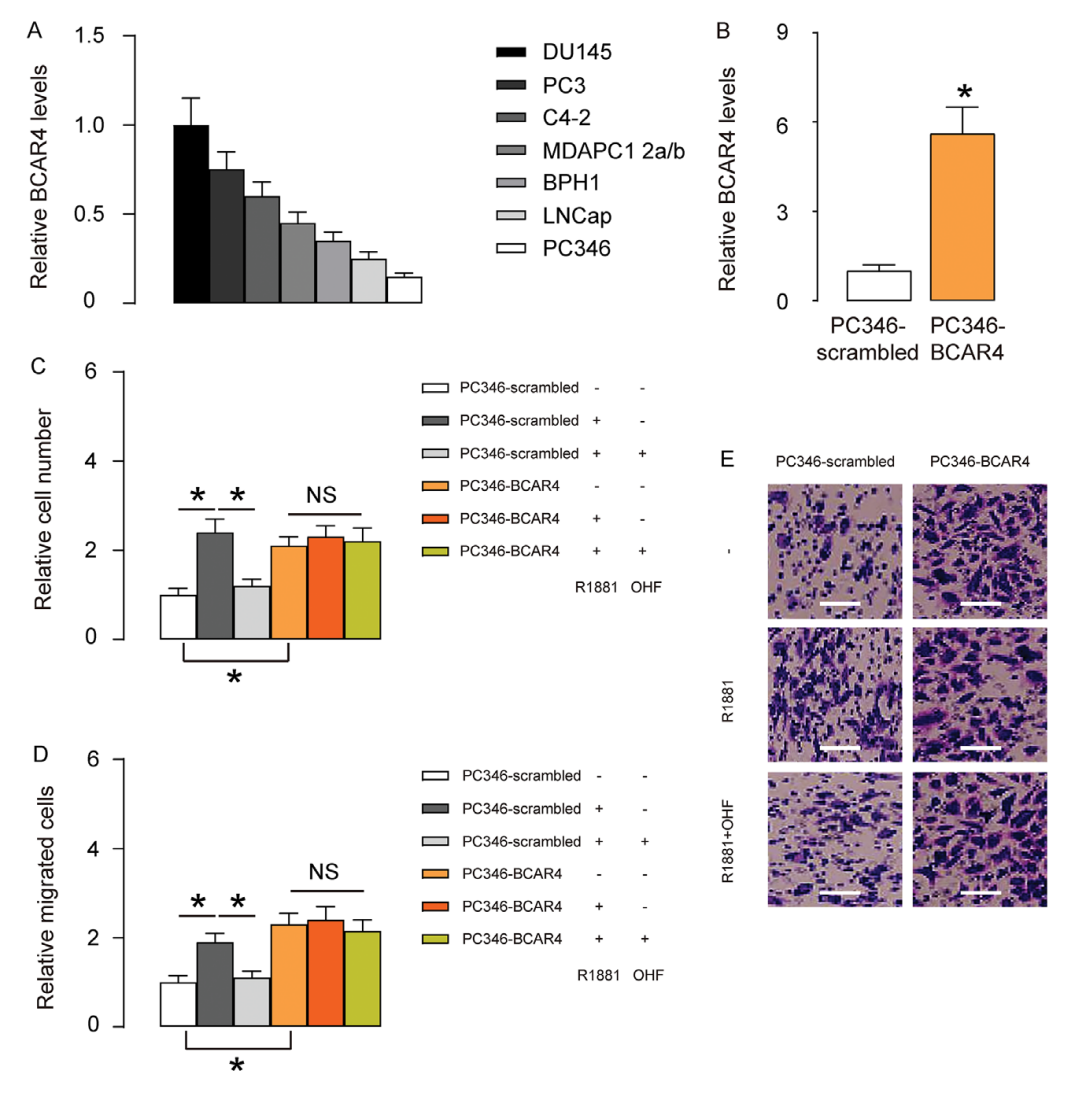
**BCAR4 increases androgen-independent PC cell growth and migration in vitro.** (**A**) RT-qPCR for BCAR4 in 7 commonly used PC cell lines. Androgen receptor (AR)- expressing PC346 expressed the least level of BCAR4, while AR-null DU145 expressed the highest level of BCAR4. (**B**) We overexpressed BCAR4 in PC346 cells, and examined BCAR4 levels by RT-qPCR. (**C-E**) BCAR4-transfected cells and control scrambled-transfected cells were treated with null, or R1881, a recombinant androgen, with/without an androgen antagonist OH-flutamide (OHF). (**C**) Cell growth was examined in an CCK-8 assay. (**D-E**) Cell migration potential was assessed in a transwell assay, shown by quantification (**D**), and by representative images (**E**). *p<0.05. NS: non-significant. N=5. Scale bars were 20µm.

Based on clinical data, we hypothesized that BCAR4 may play a role in androgen resistance in PC. To prove it, we did in vitro experiments using PC cell lines. We examined 7 commonly used PC cell lines and found that androgen receptor (AR)- expressing PC346 expressed the least level of BCAR4, while AR-null DU145 expressed the highest level of BCAR4 (Figure 2A). Thus, we overexpressed BCAR4 in PC346 cells (Figure 2B), and treated BCAR4-transfected cells and control scrambled-transfected cells with null, R1881, a recombinant androgen, with/without an androgen antagonist OH-flutamide (OHF). Cell growth was examined in an CCK-8 assay, and cell migration potential was assessed in a transwell assay. We found that androgen increased growth (Figure 2C) and migration (Figure 2D-E) of scrambled-transfected PC346 cells, both of which were abolished by OHF (Figure 2C-E). Overexpression of BCAR4 increased growth (Figure 2C) and migration (Figure 2D-E) of PC346 cells, but turned the cells insensitive to androgen (Figure 2C-E). Together, these data suggest that BCAR4 increases androgen-independent PC cell growth and migration in vitro.

### Loss of BCAR4 reduces basal PC cell growth and migration but regains androgen-sensitivity of PC cells in vitro

**Figure 3:**
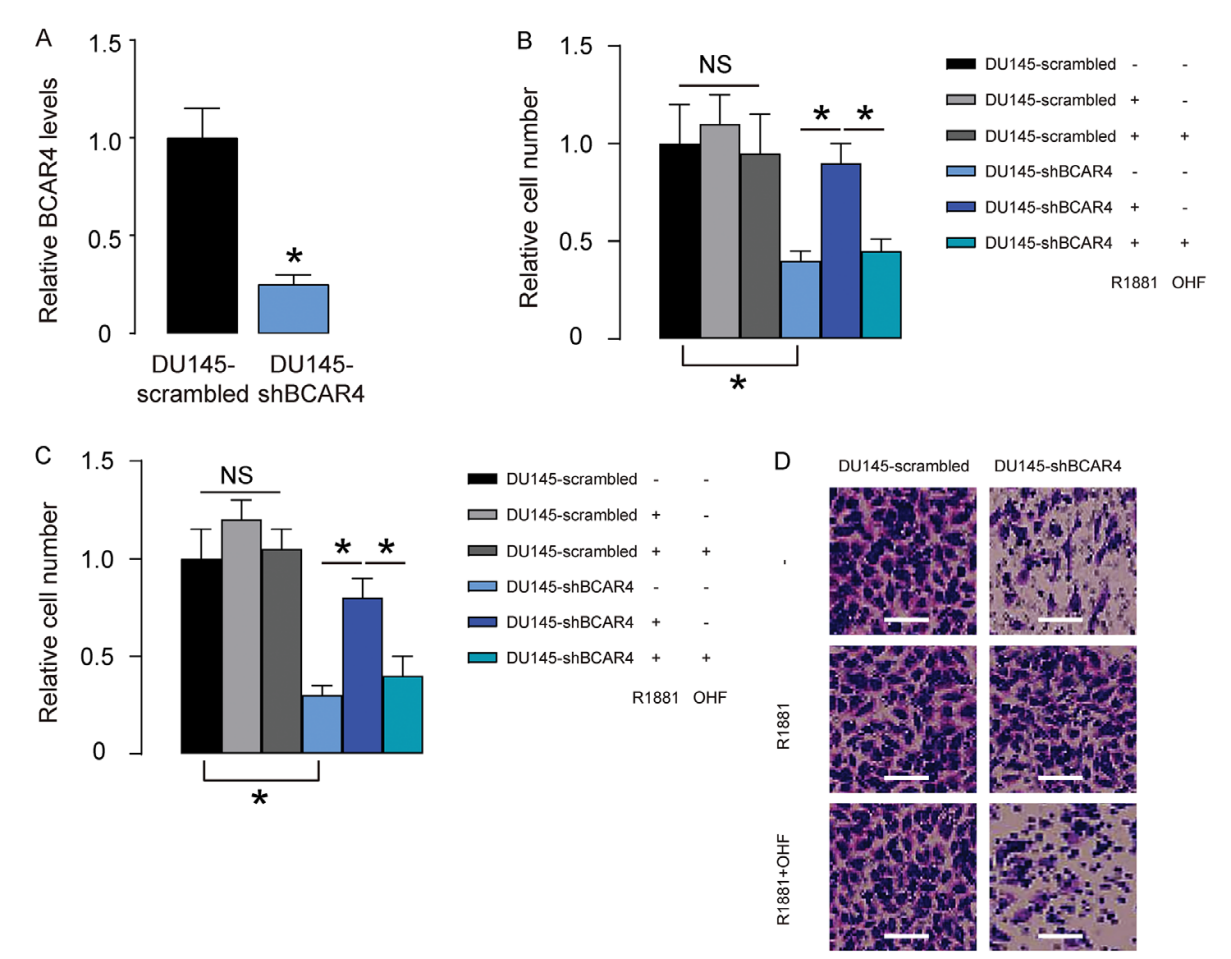
**Loss of BCAR4 reduces basal PC cell growth and migration but regains androgen-sensitivity of PC cells in vitro.** (**A**) We depleted BCAR4 in DU145 cells by shRNA and examined BCAR4 levels by RT-qPCR. (**B-D**) The shBCAR4-transfected cells and control scrambled-transfected cells were treated with null, or R1881, with/without OHF. (**B**) Cell growth was examined in an CCK-8 assay. (**C-D**) Cell migration potential was assessed in a transwell assay, shown by quantification (**C**), and by representative images (**D**). *p<0.05. NS: non-significant. N=5. Scale bars were 20µm.

Next, we depleted BCAR4 in DU145 cells by shRNA (Figure 3A), and treated shBCAR4-transfected cells and control scrambled-transfected cells with null, R1881, with/without OHF. Cell growth was examined in an CCK-8 assay, and cell migration potential was assessed in a transwell assay. We found that growth (Figure 3B) and migration (Figure 3C-D) of scrambled-transfected DU145 cells were insensitive to androgen stimulation (Figure 3B-D). Depletion of BCAR4 significantly decreased growth (Figure 3B) and migration (Figure 3C-D) of DU145 cells, which became sensitive to androgen stimulation (Figure 3B-D). Together, these data suggest that loss of BCAR4 reduces basal PC cell growth and migration but regains androgen-sensitivity of PC cells in vitro.

### BCAR4 activates GLI2-signaling pathways in PC cells

**Figure 4:**
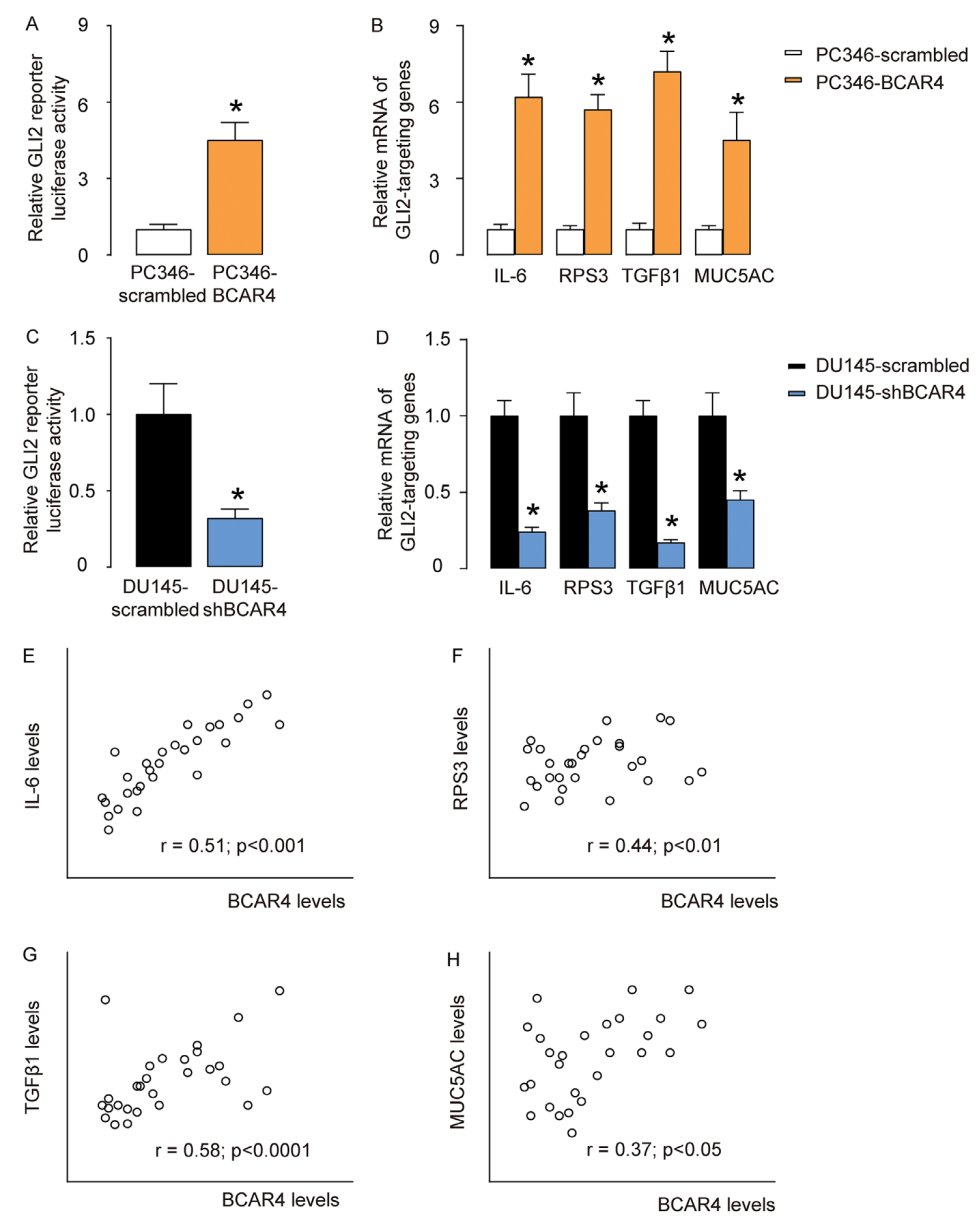
**BCAR4 activates GLI2-signaling pathways in PC cells.** (**A**) GLI2 reporter luciferase assay on scrambled or BCAR4 transfected PC346 cells. (**B**) RT-qPCR on GLI2 downstream genes IL-6, RPS3, TGFβ1 and MUC5AC in scrambled or BCAR4 transfected PC346 cells. (**C**) GLI2 reporter luciferase assay on scrambled or shBCAR4 transfected DU145 cells. (**D**) RT-qPCR on GLI2 downstream genes IL-6, RPS3, TGFβ1 and MUC5AC in scrambled or shBCAR4 transfected DU145 cells. (**E-H**) Correlation between BCAR4 levels and IL-6 (**E**), RPS3 (**F**), TGFβ1 (**G**), and MUC5AC (**H**) in the 40 CRPC specimens. *p<0.05. N=5 for A-D and N=40 for E-H.

BCAR4 has been shown binding to the promoter of GLI2 to activate GLI2 signaling pathways in breast cancer cells [16], and in osteosarcoma cells [14]. Thus, we examined if BCAR4 may also activate GLI2 signaling pathways through promoter binding. Our results showed that overexpression of BCAR4 significantly increased GLI2 reporter luciferase activity in PC346 cells (Figure 4A), and activated GLI2 downstream genes IL-6, RPS3, TGFβ1 and MUC5AC (Figure 4B). Moreover, depletion of BCAR4 significantly suppressed GLI2 reporter luciferase activity in DU145 cells (Figure 4C), and inhibited GLI2 downstream genes IL-6, RPS3, TGFβ1 and MUC5AC (Figure 4D). Furthermore, BCAR4 levels were correlated with mRNA levels of GLI2 target genes, IL-6 (Figure 4E), RPS3 (Figure 4F), TGFβ1 (Figure 4G), and MUC5AC (Figure 4H) in the 40 CRPC specimens. These data suggest that BCAR4 may activate GLI2-signaling pathways to mediate its function in CRPC.

### Depletion of GLI2 abolishes the effects of BCAR4 on growth and migration of PC346 cells

**Figure 5:**
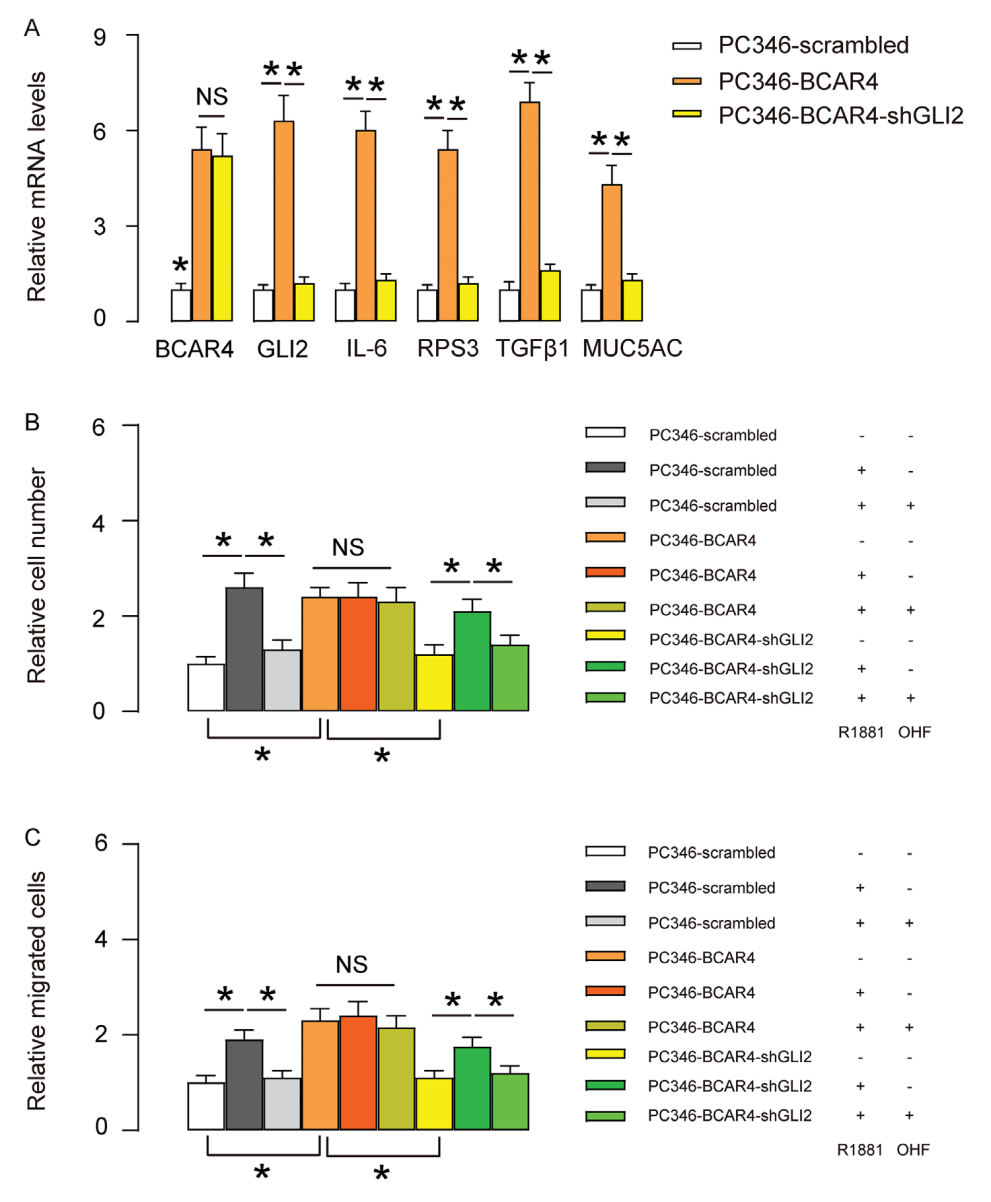
**Depletion of GLI2 abolishes the effects of BCAR4 on growth and migration of PC346 cells.** (**A**) We used shGLI2 to deplete GLI2 in BCAR4-transfected PC346 cells and did RT-qPCR for BCAR4 and GLI2. Transfection with shGLI2 significantly decreased GLI2 and GLI2 downstream genes IL-6, RPS3, TGFβ1 and MUC5AC, but did not alter BCAR4 levels. (**B-C**) These cells (shGLI2 and BCAR4-transfected cells, BCAR4-transfected cells and control scrambled-transfected cells) were treated with null, R1881, with/without OHF. (**B**) Cell growth was examined in an CCK-8 assay (**C**) Cell migration potential was assessed in a transwell assay. *p<0.05. NS: non-significant. N=5.

To assess whether the effects of BCAR4 on GLI2 target genes and PC cell proliferation and migration are dependent on GLI2, we first used shGLI2 to deplete GLI2 in BCAR4-transfected PC346 cells. We found that transfection with shGLI2 significantly decreased GLI2 and GLI2 downstream genes IL-6, RPS3, TGFβ1 and MUC5AC, but did not alter BCAR4 levels (Figure 5A), confirming that BCAR4 is upstream of GLI2. Next, these cells (shGLI2 and BCAR4-transfected cells, BCAR4-transfected cells and control scrambled-transfected cells) were treated with null, R1881, with/without OHF. Cell growth was examined in an CCK-8 assay, and cell migration potential was assessed in a transwell assay. We found that depletion of GLI2 abolished the effects of overexpression of BCAR4 on cell growth and migration in PC346 cells, and render cells sensitive again to androgen stimulation (Figure 5B-C). Thus, depletion of GLI2 abolishes the effects of BCAR4 on growth and migration of PC346 cells.

### Overexpression of GLI2 abolishes the effects of shBCAR4 on growth and migration of DU145 cells

**Figure 6:**
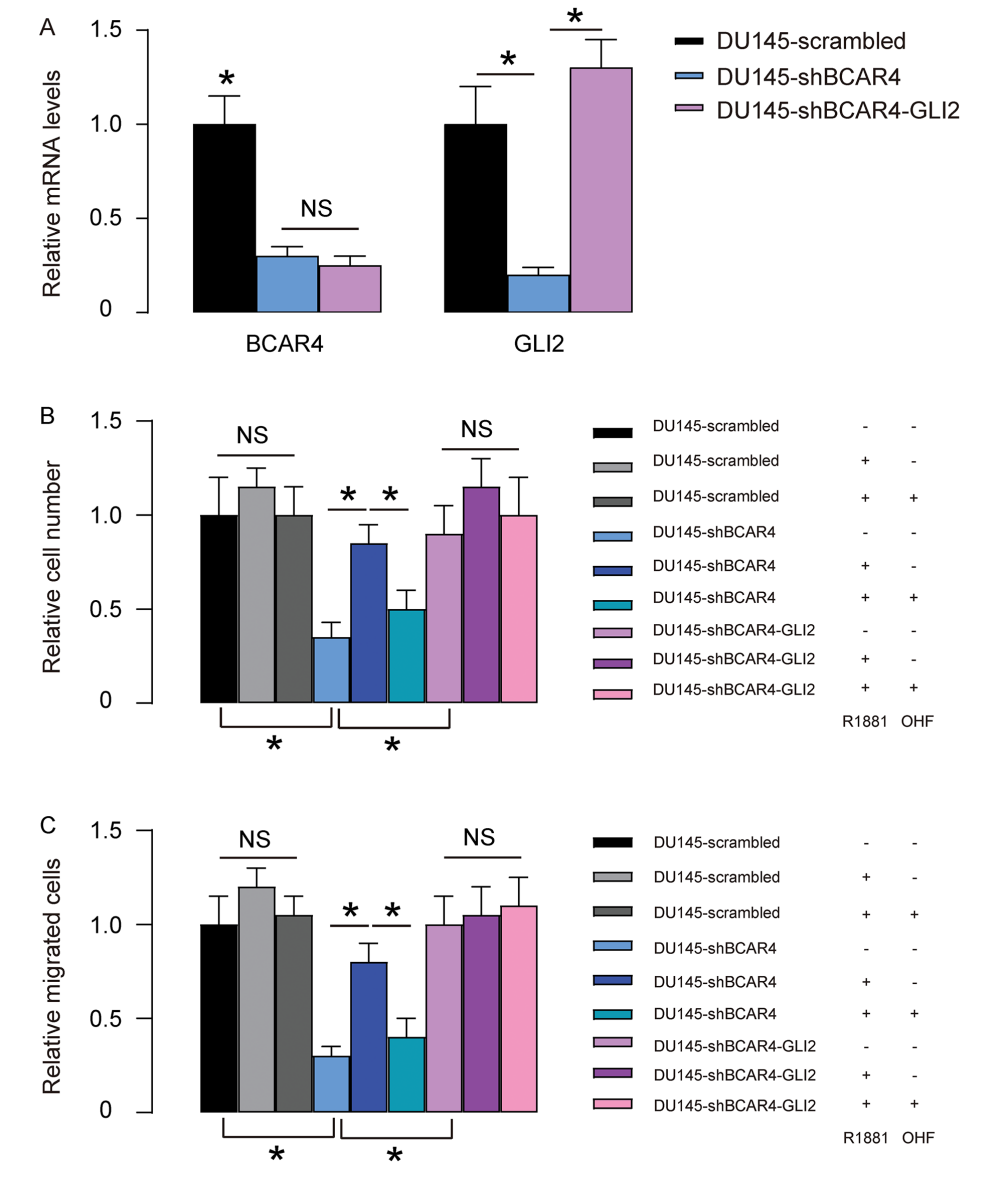
**Overexpression of GLI2 abolishes the effects of shBCAR4 on growth and migration of DU145 cells.** (**A**) We overexpressed GLI2 in shBCAR4-transfected DU145 cells and did RT-qPCR for BCAR4 and GLI2. Transfection with GLI2 significantly increased GLI2 but did not alter BCAR4 levels. (**B-C**) These cells (GLI2 and shBCAR4-transfected cells, shBCAR4-transfected cells and control scrambled-transfected cells) were treated with null, R1881, with/without OHF. (**B**) Cell growth was examined in an CCK-8 assay (**C**) Cell migration potential was assessed in a transwell assay. *p<0.05. NS: non-significant. N=5.

Next, we overexpressed GLI2 in shBCAR4-transfected DU145 cells. We found that transfection with GLI2 significantly increased GLI2 but did not alter BCAR4 levels (Figure 6A), once more confirming that BCAR4 is upstream of GLI2. Next, these cells (GLI2 and shBCAR4-transfected cells, shBCAR4-transfected cells and control scrambled-transfected cells) were treated with null, R1881, with/without OHF. Cell growth was examined in an CCK-8 assay, and cell migration potential was assessed in a transwell assay. We found that overexpression of GLI2 abolished the effects of depletion of BCAR4 on cell growth and migration in DU145 cells and render cells insensitive to androgen stimulation (Figure 6B-C). Thus, overexpression of GLI2 abolished the effects of shBCAR4 on growth and migration of DU145 cells. Together, our data suggest that BCAR4 may activate GLI2 signaling in PC to contribute to castration resistance.

## DISCUSSION

Accumulating evidence has implied several ways of lncRNA in control of development of CRPC. First, lncRNA may regulate AR signaling pathway directly. For example, Yang et al. reported that PRNCR1 And PCGEM1, two lncRNAs, can regulate the turnover of AR by forming a cyclic structure of chromatin [19]. Second, some lncRNAs may regulate the metabolism of tumor cells. For example, PCGEM1 can upregulate anaerobic glycolysis, pentose phosphate pathway, lipid metabolism and glutamine metabolism to promote tumor cell proliferation [20]. Third, lncRNAs may participate into the epigenetic regulation of tumor cells. Epigenetic changes in PC may eventually develop the cancer into castration resistance. Specially, HOTAIR, PCAT1 and ANRIL have been reported to bind to the PRC2 complex to inhibit the transcription of the AR gene itself [21]. Last, lncRNAs may interact with miRNA as a "molecular sponge" to absorb a variety of PC-related miRNAs, thereby neutralizing their tumor suppressor function. For example, CDR1as/CiRS-7 contains more than 70 binding sites for miR-7 to strongly inhibit the tumor suppressor function of miR-7 [22].

In the current study, BCAR4 was detected as a marker gene for CRPC, as its expression level was unaltered in CSPC. BCAR4 was originally found associated with tamoxifen resistance in breast cancer, in which it likely mediated the function through activating GLI2 signaling. Interestingly, control of GLI2 signaling by BCAR4 was recapitulated in CRPC cells here. From the 7 PC cell lines, we found that AR-positive cells appeared to express low levels of BCAR4, while AR-null or AR-low cells appeared to express high levels of BCAR4. These data are consistent with clinical data, showing that ARPC expressed significantly higher levels of BCAR4 than ASPC. Castration resistance is largely attributable to the expression levels of AR and the activity of AR signaling in the PC cells. Hence, BCAR4 may be associated with regulation of AR or its upstream regulator. This hypothesis was further supported by the data that overexpression of BCAR4 in BCAR4-null PC346 cells caused loss of sensitivity to androgen stimulation on cell growth and migration, and by the data that depletion of BCAR4 in BCAR4-expressing DU145 cells caused regain of sensitivity to androgen stimulation on cell growth and migration. In another word, overexpression of BCAR4 in BCAR4-null PC346 cells may lead to downregulation of AR and subsequent loss of sensitivity of the cells to androgen stimulation but activate GLI2 signaling to promote androgen-independent cell growth and migration.

The effects of BCAR4/GLI2 signaling may not only regulate AR signaling in PC, since it has androgen-independent effects on promoting PC cell growth and migration. Hedgehog/GLI2 signaling is highly conserved and regulates multiple steps in embryonic development and tissue homeostasis by controlling cell fate decisions, stem cell self-renewal, and activation. Interaction between chemokine CCL21 and its receptor CCR7 activates rho-interacting serine/threonine kinase 21, citron (CIT) [23], which phosphorylates BCAR4-activated GLI-2 at S149 to mediate its nuclear translocation [24]. A previous study has found that the expression of GLI2 was significantly higher in PC than in benign prostate hyperplasia, decreased after androgen ablation in a limited time window but became highly expressed again in CRPC [25]. Moreover, this study also showed that GLI2 appeared to play a critical role in both anti-apoptosis and growth promoting of the PC cells through modulation of apoptosis-related genes, such as Bcl2, Bcl-xL, and clusterin, and through regulation of levels of cell cycle regulators, such as cyclin D1, p27, and PKC, respectively [25]. This regulation of cell apoptosis and proliferation by GLI2 may be responsible for the BCAR4-induced androgen-independent cell growth and migration.

Collectively, our studies provided solid evidence to demonstrate BCAR4 as a prognosis factor and promising therapeutic target for CRPC.

## MATERIALS AND METHODS

### Patient specimens

This study was performed with the approval of the Ethics Committee of Shanghai Changzheng Hospital. Fifty CSPC and forty CRPC tissues with matched adjacent normal prostate tissues (NT) without prior chemotherapy or radiotherapy were obtained from patients who underwent initial biopsy at Shanghai Changzheng Hospital, after their Informed consent was obtained. All the tissues were histologically diagnosed and confirmed by two experienced pathologists. RNA was extracted from 5 pieces of each sample and kept in -70 °C. The survivals of these patients were followed up for 5 years.

### Cell culture

Seven human PC cell lines (PC346, LNCap, BPH1, MDAPC1 2a/b, C4-2, PC3 and DU145) were obtained from American Type Culture Collection (ATCC, Rockville, MD, USA). These cells were maintained in DMEM (Invitrogen, Rockville, MD, USA) suppled with 10% fetal bovine serum (FBS; Sigma-Aldrich, Rockville, MD, USA) in a humidified incubator at 37 °C with a 5% CO_2_ atmosphere. R1881 (Sigma-Aldrich) was applied to cells at a concentration of 1 nmol/l and OH-flutamide (OHF, Sigma-Aldrich) was applied to cells at a concentration of 5 µmol/l.

### Plasmids and transfection

Complete coding sequence for BCAR was obtained by PCR using human PC cell line DU145 as a template. Complete coding sequence for GLI2 was obtained from Addgene (#87097, Cambridge, MA, USA). The sequence for small hairpin RNA (shRNA) targeting BCAR4 was 5′-GGGACTTGAGTTATGTTGGTGGCTA-3′ and the sequence for shRNA targeting GLI2 was 5’-CCGCTTCAGATGACAGATGTT-3’. Scrambled shRNAs were used as negative controls. After annealing, all oligonucleotides were inserted to pCDNA3 plasmid (GenePharma, Shanghai, China). Transfection of the cells was performed using Lipofectamine 3000 (Invitrogen).

### RNA extraction and quantitative real-time polymerase chain reaction (RT-qPCR)

Total RNA was extracted with the RNeasy Kit (Qiagen, Shanghai, China) following the manufacturer’s protocol. cDNA was generated using the reverse transcription cDNA Synthesis Kit (Qiagen). The RT-qPCR was applied with the commercial SYBR Green PCR Kit (Qiagen, Shanghai, China) and the designed primers.

The primer sequences were: BCAR4: 5′-ACAGCAGCTTGTTGCTCATCT-3′ (forward) and 5′-TTGCCTTGGGGACAGTTCAC-3′ (reverse), IL6: 5′-AGACAGCCACTCACCTCTTCAG-3′ (forward) and 5′-TTCTGCCAGTGCCTCTTTGCTG-3′ (reverse), RPS3: 5′-AACTGGTAAGATTGGCCCTAAGAAG-3′ (forward) and 5′-TGTTATGCTGTGGGGACTGG-3′ (reverse), TGFβ1: 5′-TACCTGAACCCGTGTTGCTCTC-3′ (forward) and 5′-GTTGCTGAGGTATCGCCAGGAA-3′ (reverse), MUC5AC: 5′-CCACTGGTTCTATGGCAACACC-3′ (forward) and 5′-GCCGAAGTCCAGGCTGTGCG-3′ (reverse), GLI2: 5′-TGGACGTGTCCCGTTTCTCC-3′ (forward) and 5′-CCACTAGCGAGTTGGGTGAG-3′ (reverse), and β-actin: 5’-CTCTTCCAGCCTTCCTTCCT-3’ (forward) and 5’-AGCACTGTGTTGGCGTACAG-3’ (reverse). The RT-qPCR reactions were performed in duplicate. The levels of gene expression were quantified with the 2-△△Ct method and normalized sequentially with β-actin and the experimental controls.

### Cell proliferation assay

Cell proliferation was assessed using Cell Counting Kit-8 (CCK-8, Sigma-Aldrich) following the manufacturer’s instructions. Briefly, a total of 1500 cells were seeded in each well of 96-well plates. Cell viability was measured using the absorbance at 450 nm at 72 hours after seeding. All experiments were performed in duplicate.

### Cell migration assay

Cells were suspended in serum-free medium and then seeded in the top chamber of a 24-well transwell insert (Millipore, Bedford, MA, USA). The lower chamber was filled with medium supplemented with 10% FBS. After 48 hours, the cells that had migrated to the lower surface were fixed with methanol, stained with crystal violet, and counted.

### Luciferase reporter assay

GLI2 luciferase reporter plasmid (Promega, Madison, WI, USA) was transfected into cells using Lipofectamine 3000 (Invitrogen), followed by analysis with Dual-Glo Luciferase Reporter Assays (Promega). The relative luciferase activity was measured and quantified 48 hours after transfection.

### Statistical analysis

The data was statistically analyzed with GraphPad Prism 7 (GraphPad, Chicago, IL, USA). The Student’s T test was performed to compare the data of 2 groups. The values were expressed as mean ± standard deviation (SD). When p<0.05, the data was considered as significant. Bivariate correlations were calculated by Spearman's Rank Correlation Coefficients. Kaplan-Meier curve was applied to record the overall survival of the patients included in this study.
